# Effect of Major Mycotoxins on Public Health Through the Consumption of Cheese Products

**DOI:** 10.21315/mjms2024.31.6.3

**Published:** 2024-12-31

**Authors:** Elias Ath. Chaidoutis, Olympia Chatzimpirou, Ioannis Migdanis, Athanasios Migdanis, Antonios Papadakis, Andreas Ch. Lazaris, Nikolaos Kavantzas

**Affiliations:** 1 First Department of Pathology, School of Medicine, National and Kapodistrian University of Athens, Athens, Greece; 2 Department of Gastroenterology, Faculty of Medicine, University of Thessaly, Larissa, Greece; 3 Department of Nutrition and Dietetics, University of Thessaly, Trikala, Greece; 4 Department of Clinical Microbiology and Microbial Pathogenesis, School of Medicine, University of Crete, Heraklion, Greece

**Keywords:** mycotoxins, aflatoxin M1, ochratoxin A, cheese products, public health

## Abstract

Food safety is a key priority for public health. However, consumer demand for cheese products may expose the population to the risk of mycotoxicosis and cancer, among others. Acute mycotoxicosis and cancer are examples of linked disorders. Among the most frequent toxic agents that enter the human body through food consumption are mycotoxins. This review study highlights the significance of the impact of the most important mycotoxins on public health through the consumption of cheese products. Despite being a poor substrate for mycotoxin development, cheese products have been found to contain harmful toxins. Aflatoxin M_1_ (AFM_1_) and ochratoxin A (OTA) are the main mycotoxins in cheese products, and they are very harmful to human health. Adherence to legislative limits and the implementation of appropriate control measures by food business operators (FBOs) are considered necessary to protect consumers’ health.

## Introduction

Food safety plays a key role in public health. Chemical compounds in food cause global concern for consumer health and constitute a main cause of trade barriers. Chemical compounds found in food may be either intentionally added for technological purposes (e.g., food additives) or through environmental air, water, and soil pollution (1).

Mycotoxins are toxic compounds that are naturally produced by certain types of moulds (fungi). Moulds that can produce mycotoxins grow on numerous foodstuffs, such as cereals, dried fruits, nuts, and spices. Mould growth can occur either before or after harvest and during storage under warm and humid conditions (2). It is estimated that 25% of global crop production is contaminated with mycotoxins (3). The effects of mycotoxins transmitted through the consumption of contaminated food may be acute, with symptoms of severe illness. Other mycotoxins transmitted indirectly from animals that are fed contaminated feed, in particular from milk, have been linked to long-term effects on health, including the induction of cancers and immune deficiency (2, 4). Several hundred different mycotoxins have been detected so far, with aflatoxins and ochratoxins the most common (2).

Milk and dairy products are major sources of human nutrition (5). Cheese products have been among the basic foodstuffs since ancient times (6, 7). As one of the most commonly used foods, they are in high demand by consumers (8). Greece has a long tradition of cheese production and consumption, and it is ranked high in cheese consumption globally (9). It is among the countries with the highest number of cheese types produced, along with France, Germany, Italy, and the Netherlands (10). Cheese products are a sensitive product in terms of mould formation (11). Aflatoxin M_1_ (AFM_1_) and ochratoxin A (OTA) are recognised as the most dangerous mycotoxins (12, 13). The purpose of the present review is to highlight the significance of the impact of AFM_1_ and OTA on public health through the consumption of cheese products.

## Mycotoxin Formation in Food

Mycotoxins are a group of secondary metabolites produced mainly by several filamentous fungi (14, 15). They are low molecular weight organic compounds, and they are toxic for people, animals, plants, and microorganisms, even in low concentrations (15, 16). The term mycotoxins originates from the Greek word “μύκης” (mykes), which means fungus, and the Latin word “toxicum”, which means poison (17).

Mycotoxins were first identified in 1960 in the aftermath of an unprecedented veterinary crisis near London (Turkey X disease), during which approximately 100,000 turkey poults died (18, 19). Soon afterwards, the use of the term “mycotoxins” was expanded to include not only the known fungal toxins, as well as several new fungal secondary metabolites that appeared later (e.g., OTA), but also other compounds that had originally been isolated as antibiotics (14, 15, 20). Mould growth on certain foodstuffs and feed under favourable environmental conditions has resulted in the formation of mycotoxins (21), which are produced sporadically under fungal stress. Most mycotoxins in feed and foodstuffs are produced by the fungal genera *Aspergillus*, *Penicillium*, and *Fusarium* (3, 22). Some mycotoxins that have been found in foodstuffs include aflatoxin, patulin, fumonisin, ochratoxin, ergotamine, zearalenone, t-2 toxin, cyclopiazonic acid, and sterigmatocystin (23). Aflatoxins, OTA, fumonisins, and zearalenone have been identified as among the most important mycotoxins (24, 25).

Endogenous and exogenous factors can influence the formation of fungi that produce mycotoxins. Endogenous factors include water activity (aw), pH, and redox potential. Exogenous factors include relative humidity, oxygen sufficiency, and temperature (21). The two basic reasons for their production are their competition with other microorganisms for nutrient content and the development of favourable conditions for their seeds to grow (21, 26).

Generally, mycotoxins are found more often in feed (cereals, maize, rice, cottonseed, peanuts, legumes, and barley) and foodstuffs of plant origin (27). They can also be detected in foodstuffs of animal-origin, such as meat (and intestinal organs), milk, and eggs. Their presence in these foodstuffs is usually at lower levels than in feed (20).

## Toxic Effects of Mycotoxins on Human Health

A major source of people’s exposure to mycotoxins is the consumption of contaminated foodstuffs. The illnesses caused by exposure to mycotoxins are known as mycotoxicoses (15, 28). Exposure through the consumption of foodstuffs can cause a variety of toxic effects, including haemorrhagic, hepatotoxic, nephrotoxic, neurotoxic, oestrogen, teratogens, immunosuppressant, mutagenic, and cancer effects (29). Aflatoxins, ochratoxin, sterigmatocystin, patulin, cyclopiazonic acid, citrinin, and other mycotoxins found in foodstuffs have been classified by the International Agency for Research on Cancer (IARC) as carcinogenic substances (30, 31).

Mycotoxins can be immunotoxic, but this depends on the level of exposure (32, 33). The severity of mycotoxicosis also depends on biological condition, gender, age, and length of exposure to the respective mycotoxin (34). Mycotoxins have various acute and chronic effects on people and animals (15, 29). In acute mycotoxicoses, symptoms in the human population appear rapidly. Chronic mycotoxicoses are a result of long-term, low-dose exposure to mycotoxins, which can lead to cancer and other irreversible toxic effects. In humans and animals, contamination with mycotoxins is usually associated with chronic exposure to mycotoxins (e.g., cancer, kidney toxicity, immunosuppression, etc.) (30, 33).

Human exposure to mycotoxins can be further determined by environmental monitoring and bio indices (30). Several reports have shown that mycotoxins, such as aflatoxin, ochratoxin, and patulin, can influence the inflammatory response (35).

## Mycotoxins in Cheese Products

The most common mycotoxins found in various types of cheese products are aflatoxins, OTA, sterigmatocystin, penicillic acid, patulin, Penicillin Roquefort (PR) toxin, roquefortine, citrinin, cyclopiazonic acid, and mycophenolic acid (36, 37). Besides the presence of mycotoxins with carcinogenic effects (AFM_1_), the presence of mycotoxins with nephrotoxic effects (OTA) in cheese products has also been reported. Other mycotoxins, such as patulin and penicillic acid, have also been reported, but they are not stable in cheese products (16).

Mycotoxins can be found in cheese products either because they pre-exist in milk (AFM_1_, AFM_2_), or because they are formed during their ripening and preservation processes following intense surface fungal growth (ochratoxin, patulin, cyclopiazonic acid, roquefortine, etc.). Contamination can also occur naturally from the equipment used or from the air (38). The presence of fungi in cheeses is harmful and undesired in most types of cheese, apart from mould-ripened cheeses (e.g., blue cheeses, Camembert, and Brie), in which the presence of fungi is desired (39, 40). The potential presence of mycotoxins in certain fungal strains of the *Penicillium* genus involved in cheese ripening (PR toxin, cyclopiazonic acid, roquefortine, etc.) has also been examined (41).

The *Aspergillus* and *Penicillium* genera are among the most common fungal genera to produce mycotoxins in cheese products (Table 1).

The fungal species of the *Penicillium* genus can also grow on cheese at cooling temperatures, but the fungal species of the *Aspergillus* genus need higher temperatures to grow (16). The presence of mycotoxins in cheese is due to three main factors:

- The presence of aflatoxin M1 (AFM_1_) in fresh or processed milk that is used in the production of cheese, arising from feed contaminated with aflatoxin B1 (AFB_1_) that has been consumed by dairy cattle. This is an indirect method of cheese contamination.
- The production of mycotoxins by fungal species, such as *Aspergillus* and *Penicillium*, that grow on cheese constitutes a direct method of cheese contamination.
- The production of mycotoxins by fungal species used to produce mould-ripened cheeses is another direct way of producing cheese contaminated with mycotoxins.

The formation of wild fungal strains on the surface of cheese has been studied in numerous experiments to determine how deep into the cheese mycotoxins can penetrate. Several studies in which toxigenic fungi have been injected into cheese have shown aflatoxin formation, and some experiments have revealed the formation of OTA and other mycotoxins (41). Fungal penetration depth may be limited by monitoring the availability of oxygen required for the production of fungi (38). Several mycotoxins that have been produced by fungal growth on cheese have been reported to penetrate cheese masses 1 cm–4 cm deep (41).

Hard cheeses are of particular interest regarding fungal growth on their surfaces. Only a limited number of these cheeses produce mycotoxins (2%–15%), which is probably due to the low concentration of carbohydrates and the high concentration of proteins (38). The most important factors influencing mycotoxin production in cheese products are temperature and relative humidity, which are possibly low if cheese is stored in cooling conditions. The most common fungi that contaminate hard cheeses are species of the *Penicillium* genus (>80%) that grow easily in cooling conditions. The species of *Aspergillus* genus can also contaminate the hard surface of cheeses (<8%), but they do not grow, and they do not produce toxins in temperatures below 10°C–13°C. Mycotoxins in cheese products can withstand high temperatures. The manufacture of certain cheeses usually includes the heat treatment of the cheese and its ingredients. Cheese powders used in cheese-making may also undergo heat treatment. The temperature levels may reach up to 105°C for 60 seconds. However, it has been demonstrated that several types of mycotoxins remain relatively stable during most of the heat treatments in use today (38).

## Aflatoxins

Aflatoxins constitute one of the most important groups of toxic agents in foodstuffs and feeds (42). The fungal species of *Aspergillus flavus*, *Aspergillus parasiticus*, and *Aspergillus nonius* that produce aflatoxins grow in increased relative humidity, which usually amounts to more than 85% (43, 44). Apart from temperature, the water activity coefficient (aw) and the presence of nutrients also affect the conditions that favour fungal growth (45). *Aspergillus flavus* produces aflatoxin B while *A. parasiticus* produces aflatoxins B and G. Almost half of *A. flavus* strains produce aflatoxins (43). *Aspergillus parasiticus* may produce aflatoxins at temperatures of 6°C–46°C (optimum production temperatures are 25°C–35°C). *Aspergillus flavus* may produce aflatoxins at temperatures of 12°C–42°C (optimum values are 28°C–30°C) (43).

Approximately 20 different types of aflatoxins have been identified. The four main types are B_1_ (AFB_1_), B_2_ (AFB_2_), G_1_ (AFG_1_), and G_2_ (AFG_2_) which are usually found in foodstuffs (23). Aflatoxins AFB_2_ and AFG_2_ are hydroxylated derivatives of the aflatoxins AFB_1_ and AFG_1_. Following the consumption of contaminated feed, AFB_1_ is metabolised in the livers of animals. AFM_1_ is the main hydroxylated metabolite of AFB_1,_ and it is derived from milk-producing animals that have consumed feed contaminated with AFB_1_ (16). AFM_1_ is approximately 10 times less toxic for humans than AFB_1_ (46). The dose quantity, overall time of exposure, and the way they are metabolised are the most important factors influencing the toxic effect of aflatoxins (47).

Aflatoxicosis is the most important foodborne disease caused by aflatoxin in both animals and humans (5, 48). Exposure to high doses of aflatoxins may lead to acute intoxication and may be life threatening, usually by damaging the liver (2, 44). Foodstuffs from animals with chronic subclinical toxicities are contaminated with aflatoxins and therefore can put consumers’ health at risk. Cases of acute aflatoxicosis in humans have been seen occasionally in some countries, mainly in the developing countries of Africa and Asia (49). In developing countries, an estimated 4.5 billion individuals are exposed to aflatoxin, which is one of the major causes of liver cirrhosis and hepatocellular carcinoma (HCC). In 2013, some countries in Europe, including Romania, Serbia, and Croatia, reported that milk was contaminated nationwide with aflatoxin (50).

**Figure 1:**
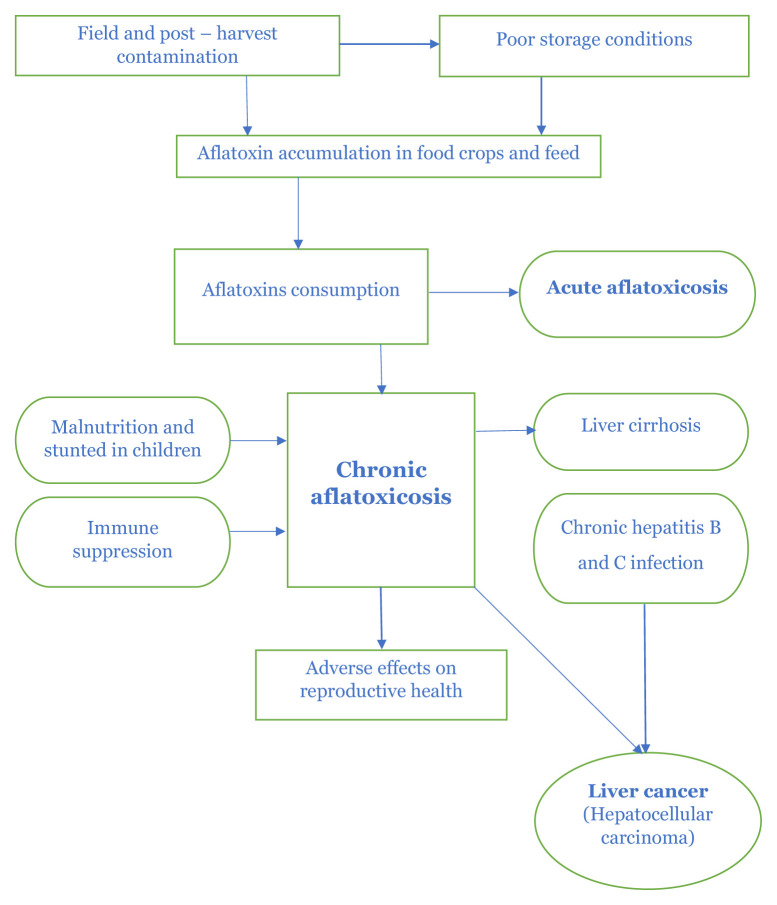
Routes of exposure to aflatoxins in humans (Adopted by Bbosa et al. 2013) (55)

Exposure to aflatoxin has an especially negative effect on children. Several studies have indicated a link between childhood aflatoxin exposure and development retardation. Adults are more resistant to aflatoxin exposure than children. Exposure to aflatoxin has been linked to HCC, accounting for 4.6% to 28.2% of HCC occurrences worldwide. Death may result from acute high-dose exposure in about 25% of cases. The majority of presenting cases of aflatoxin toxicity are late presentations after chronic exposure. Acute high-dose exposures are rare, and the most severe cases occur in children (51). The chronic forms result from low aflatoxin intake through food consumption over a long time (52). Epidemiological studies have demonstrated a positive correlation between aflatoxins and foodstuffs as a causative agent for the induction of primary HCC, while there is evidence of the synergistic action of aflatoxins in the effects of the hepatitis B virus (28). In 1993, the IARC classified the aflatoxins AFB_1_ and AFM_1_ as 1 (human carcinogens) and 2b (probable human carcinogens), respectively (28, 30, 44). It is estimated that exposure to aflatoxins may be responsible for 5%–28% of all cases of hepatocellular cancer worldwide (41, 42). Milk is a foodstuff that contains AFM_1_ more often than other animal-origin foodstuffs (53) and can cause aflatoxicosis which is the most important foodborne disease through the consumption dairy products (5). In developed countries, aflatoxin exposure is not a major problem due to strict regulatory requirements and more diverse diets than in developing countries (54). The routes of exposure to aflatoxins in humans are illustrated in Figure 1 (55).

### Occurrence of AFM_1_ in Cheese Products

The amount of AFB_1_ in the feed that is excreted in the form of AFM_1_ in cow’s milk is approximately 1%–3%, but values of up to 6% have been reported (56). It has been estimated that if a cow consumes a quantity of 300 ng/g AFB_1,_ it will produce milk containing 1–3 ng/mL AFM_1_ 24 hours later. The AFB_1_ to AFM_1_ conversion ratio can vary from 1:100 to 1:300 (16). Similar conversion ratios of AFB_1_ in feed to AFM_1_ in milk are also seen in sheep and other milk-producing animals (57).

AFM_1_ is the most frequently reported mycotoxin in cheese worldwide (44). A study in Egypt revealed that AFM_1_ was found in 2 of 13 local soft cheese samples manufactured from naturally contaminated milk at levels that exceeded the legislative limits of the European Union. Cheese products manufactured from AFM_1_-contaminated milk showed a higher concentration of mycotoxin in cheese curd than in manufactured yogurt (48). Compared with other dairy products, cheese is a potential source of AFM_1_ due to the presence of casein, which is highly concentrated during its manufacture (7, 48). In another study based on data from various countries (Czech Republic, Slovakia, France, Greece, Germany, Iran, Switzerland, Syria, Holland, etc.), the prevalence of AFM_1_ in cheese varies (58). In a study conducted in southern Spain based on 35 samples of local cheese, AFM_1_ was detected in 16 (44.7%) samples in concentrations between 20 and 200 μg/g (16). The type of cheese may play a role in the concentration of mycotoxins. Quantitative data on the concentration of AFM_1_ in some types of cheeses made with milk from cows and goats were evaluated through a global review conducted by Khaneghah et al. (10), which concluded that fresh cheeses contained a higher level of aflatoxins compared with other types of cheeses. In a study conducted in Turkey, the presence of AFM_1_ was detected in 75 fresh and fermented cheese samples (71.42%) out of 105 samples analysed. In addition, the levels of AFM_1_ in 40 cheese samples (38.08%), a large proportion of which were fresh cheeses (59), were found to exceed the allowed national legislative limits (250 ng/kg). AFM_1_ remains stable in milk and cheese products (36). A strong association of AFM_1_ with casein is referred (60). Most researchers agree that cheeses contain 40% to 60% of the AFM_1_ that pre-exists in milk, and the rest of the AFM_1_ is found in whey (61); 45%–50% of AFM_1_ and AFM_2_ penetrate the curd during cheese-making. However, due to the concentration procedure, the concentration of AFM_1_ and AFM_2_ in the curd is 3.5 to 5.0 times greater than that in the milk from which the cheese is produced (41).

Besides the indirect presence of aflatoxins (AFM_1_) in cheese, there is a direct presence. The ability of toxicogenic *Aspergillus* to produce other types of toxins besides AFM_1_ has been observed in experimental studies. In a study that used different types of cheddar cheese, the results showed that aflatoxins can penetrate 4 cm deep into the cheese surface (62). Similar results were observed in earlier experimental studies, such as Frank (1968), who grew *A. flavus* on Tilsit cheese; 200 μg/kg of AFB_1_ 0.5 cm deep into the surface of the first slice of cheese and 600 μg/kg of AFG_1_ were observed. The aforementioned aflatoxins were also found 1 cm deep into the cheese surface, but in much smaller quantities (20 μg/kg) (63). Comparisons between studies that used strains of *A. parasiticus* demonstrated lower production of aflatoxins in cheese products regarding the presence of *A. flavus*. In research conducted to identify aflatoxins in processed cheeses that were contaminated with *A. flavus*, aflatoxins AFB_1_, and AFG_1_ were detected in quantities that diminished gradually depending on the penetration depth from the surface of the cheese (64). In a Greek study on feta cheese injected with *A. Flavus* fungi, aflatoxins AFB_1_ and AFG_1_ were found on the third surface layer of the cheese (0.24 cm) the length of each layer was determined by the researcher at 0.8 cm were found in much smaller quantities than in the first surface layer of the cheese (0.9%–1.5%)(53).

## Ochratoxin A

One of the most significant and harmful mycotoxins is OTA (65). It is considered the most prevalent and most toxic of all the ochratoxins (40). OTA can be produced from various species of the fungal strains *Aspergillus* and *Penicillium* and others (14). It constitutes one of the most common toxic agents in foodstuffs (66). OTA and the metabolites thereof are produced in foodstuffs, mainly from the species *Penicillium verrucosum* and *Aspergillus ochraceus* (20). *Penicillium verrucosum* is the dominant producer of OTA; however, *Penicillium nordicum* can also produce it (65). The fungus grows more often on foodstuffs of plant origin, but it can also be found in food products with a high protein and fat concentration, such as meat and cheese (67).

OTA is related to chronic kidney disease, which is defined as Balkan endemic nephropathy (BEN) (40, 68). The phenomenon of OTA-induced nephrotoxicity in humans is considered to play a key role in the aetiology of neuropathies and tumours of the urinary system (69). In addition to nephrotoxicity, research has shown that OTA can be toxic to the liver and a key regulator of the immune system (32, 70). According to the European Food Safety Authority’s (EFSA) risk assessment regarding OTA in foodstuffs, based on data on the mechanisms of OTA toxicity, weekly intake must not exceed 120 ng/kg body weight (71). In 1993, the IARC classified OTA into category 2b (probable human carcinogens) (28, 30, 68). Following the digestion of feed contaminated with ochratoxins, OTA is converted into a less toxic metabolite ochratoxin α. OTA and the metabolites thereof are usually detected in the urine and faeces of animals, but they have also been detected in their milk (72, 73). The high toxicity of OTA is a major health concern that has led to an increased need for accurate monitoring of this mycotoxin in food products (74).

### OTA in Cheese Products

In addition to aflatoxins, there is evidence of the presence of OTA in cow’s milk. A study in Italy investigated the presence of OTA in 73 samples of various types of cheese purchased from local markets. Sixty-nine of the samples were below the limit of quantification (LOQ), while four samples showed an OTA concentration level ranging from 1.3 to 7.5 pg/kg, indicating that there was a low risk of OTA contamination. All of the positives were samples of grated hard cheese made from cow’s milk (40). In another study conducted in Sweden regarding the presence of OTA in cow’s milk, the results showed the presence of mycotoxin in 14% of the 36 samples, in quantities ranging from 10 to 40 ng/mL of milk (72). In another study in Italy, 22 samples of cave-ripened cheeses were surveyed for their mycotoxigenic potential in vitro and mycotoxin content in cheese products, and the researchers concluded that the intake of OTA from cheese seems to be of limited significance for the general population (75). Experimental studies have focused on the prevalence of OTA in cheeses. In a study on the transfer of OTA in sheep’s milk used for the manufacture of cheese products, the amount of the toxin transferred to milk was determined to be lower than 1% (73). Another study conducted by Kokkonen et al. (76) concluded that cheese products do not constitute an appropriate substrate for the surface growth of fungi that produce OTA (e.g., *P. verrucosum*). A study on the occurrence of mycotoxins concerning the surface growth of fungi on cheddar cheeses concluded that significant quantities of OTA were detected 0.7 cm deep into the surface of the cheese sample. OTA has been reported to be relevantly stable in cheddar cheeses; 49% of OTA appears to be preserved in storage conditions at 25°C for 48 hours (37). In France, Comte semi-hard ripened cheese was examined after artificial inoculation with an OTA-producing *P. verrucosum* strain. At 8°C, OTA production started after 28 days of incubation, while at 20°C, mycotoxin was produced from the seventh day. The maximum OTA concentration was approximately 4,000 ng/g of cheese. Maximum concentrations were obtained in the top part of the cheese, but the mycotoxins were up to 1.6 cm deep (77). The results of a study in Germany on a quantification method for determining the toxicology of mycotoxin OTA applied for a market screening of hard, semi-hard, and soft cheese samples shown that two samples of hard cheese with particularly high OTA levels of 4.8 pg/kg and 27 pg/kg were identified (78). In addition, a survey conducted to investigate the presence of OTA in cheese and pork meat products, based on data from official control sources, concluded on the basis of an analysis of 75 samples that the occurrence of OTA was quite low and only occasional in the food considered. However, the detection of mycotoxin in concentrations above LOQ (1 pg/kg) in grated cheeses poses a possible risk to the health of consumers (79). The results of this study support the findings of an earlier study conducted in Italy by Biancardi et al. (80), in which OTA was detected in six out of 40 commercially grated hard cheeses at high levels that can be harmful for consumers health.

## Mycotoxin Control Measures and Legislative Levels in the European Union

To control mycotoxins, it is necessary to study and understand the factors that allow fungi to grow and produce mycotoxins (81). Mycotoxin production depends on various factors, such as water activity, temperature, substrate, fungal strain, presence of chemicals, and microbial interactions (81). The most dangerous mycotoxins do not usually grow to a significant extent on low-carbohydrate foods, such as cheese, in under-ripening conditions (82).

AFM_1_ may concentrate in the cheese-making process, although its preservation in the curd depends on the technology used. Avoidance of contaminated feed remains the main control measure (82). The surface growth of the relative fungal species can be controlled by three basic measures: surface protection, limited access to oxygen, and low temperature. Table 2 summarises some of the mycotoxin control measures used with cheese products. Cheese products in which fungal growth is visible can be used for further processing to the extent that measures are adopted to control these fungi in a way that prevents the development of mycotoxins. It is worth noting that when cheese products are combined with seasonings (herbs, flavourings, flavour enhancers, etc.), a specific assessment must be carried out to determine whether additional types of fungi have penetrated, which are likely to produce mycotoxins, and if any extra controls will be needed to ensure that the possibility of mycotoxin formation in these cheese products is minimised (83).

Only a few species of fungi produce toxins at low temperatures, and the concentration of these mycotoxins in cheese is influenced by many factors. It has generally been acknowledged that relative humidity and temperature are considered the most critical factors. Storage in cool temperatures, in combination with vacuum or modified temperature (MAP) packaging, for example, which provides a relatively high concentration of dioxide (>50%) or/and low concentration of residual oxygen (<0.5%), will prevent fungal growth on cheese. Finally, the surface removal of fungi visible at a penetration depth of 4 cm is sufficient to remove any mycotoxins that may exist (83).

The European Regulation (EU) 2023/915 requires that the maximum level of AFM_1_ in milk, heat-treated milk, and milk intended for the processing of cheese products be 0.050 μg/kg (84). For the determination of the maximum permissible level in cheese products, concentration and dilution criteria apply (82). In Regulation (EC) 1881/2006, which has been repealed by Regulation (EU) 2023/915, it is explicitly stated that even if AFM_1_ is considered a less dangerous, genotoxic, and carcinogenic substance, it is necessary to avoid its presence in milk and other dairy products, especially in those intended for young children (85, 86).

## Discussion

The levels of mycotoxins reported in the published literature on cheese products are quite low. The expectation that cheese products are usually consumed in small quantities reduces the risk to human health. Nevertheless, even at low levels, AFM_1_ in cheese products raises concerns about public health. Cheeses can be susceptible to OTA contamination if the milk used in their production is contaminated with OTA-producing moulds. Some researchers have concluded that cheese products are not a suitable substrate for the growth of OTA-producing fungi. However, various experimental studies on the surface growth of fungi have observed OTA on cheese surfaces. In addition, the research findings on cheese products that are widely available in the marketplace, especially grated hard cheeses, are of particular interest.

## Conclusion

Mycotoxins are among the most common toxic substances transferred into the human body through the consumption of foodstuffs. Cheese products are considered a poor substrate for mycotoxin production; however, the presence of dangerous toxins in them has been reported. Among the most important mycotoxins for human health through the consumption of milk are AFM_1_ and OTA.

AFM_1_ is the main mycotoxin in cheese products; however, its direct effect on the development of liver cancer through the consumption of cheese products requires further research. Regarding OTA, there is insufficient evidence to determine its transfer rate in cow’s milk. Further research is needed to draw sounder conclusions about the production of OTA from mould growth on the surface of cheese. The legislative limits established by the European Union for the maximum allowable limits for AFM_1_ in dairy products help to minimise the risk to consumers. Strict adherence to preventive measures by food business operators (FBOs), such as Hazard Analysis Critical Control Point (HACCP), is also considered very important to prevent the presence of mycotoxins in cheeses.

## Figures and Tables

**Table l:** The most important fungal genera that produce mycotoxins in cheese products

Genus *Penicillium*	Genus *Aspergillus*
OTA (*Penicillium verrucosum*)	AFM_1_
Patulin	OTA (*Aspergillus ochraceus*)
Citrinin	OTA (*Aspergillus niger*)
Penicillanic acid	Sterigmatocystin
Cyclopiazonic acid	
PR toxin	
Roquefortine	
Mycophenolic acid	

Source: O ‘Brien et al. (16)

**Table 2:** Mycotoxin control measures in cheese products

Site of production	Implementation of control measures	Reference
Refrigerated storage	Temperatures below 9°C	(83)
Packaging	Low oxygen and/or high concentration of other gases	(81)
Production	Addition of competitive microorganisms	(81)
Production	Addition of natamycin	(89)
Production	Use of potassium sorbate in cheeses 0.2%–0.3%	(90)
Production	Addition of natural plant extracts	(91)
Production	Regulation of acidity (pH value approximately 4)	(92)
